# 7T ultra-high-field neuroimaging for mental health: an emerging tool for precision psychiatry?

**DOI:** 10.1038/s41398-022-01787-3

**Published:** 2022-01-26

**Authors:** Irene Neuner, Tanja Veselinović, Shukti Ramkiran, Ravichandran Rajkumar, Gereon Johannes Schnellbaecher, N. Jon Shah

**Affiliations:** 1 Institute of Neuroscience and Medicine 4, INM-4, Forschungszentrum Jülich, Jülich, Germany; 2 Department of Psychiatry, Psychotherapy and Psychosomatics, RWTH Aachen University, Aachen, Germany; 3 JARA-BRAIN, Jülich/Aachen, Germany; 4 Department of Neurology, RWTH Aachen University, Aachen, Germany; 5 Institute of Neuroscience and Medicine 11, INM-11, Forschungszentrum Jülich, Jülich, Germany

**Keywords:** Prognostic markers, Depression

## Abstract

Given the huge symptom diversity and complexity of mental disorders, an individual approach is the most promising avenue for clinical transfer and the establishment of personalized psychiatry. However, due to technical limitations, knowledge about the neurobiological basis of mental illnesses has, to date, mainly been based on findings resulting from evaluations of average data from certain diagnostic groups. We postulate that this could change substantially through the use of the emerging ultra-high-field MRI (UHF-MRI) technology. The main advantages of UHF-MRI include high signal-to-noise ratio, resulting in higher spatial resolution and contrast and enabling individual examinations of single subjects. Thus, we used this technology to assess changes in the properties of resting-state networks over the course of therapy in a naturalistic study of two depressed patients. Significant changes in several network property measures were found in regions corresponding to prior knowledge from group-level studies. Moreover, relevant parameters were already significantly divergent in both patients at baseline. In summary, we demonstrate the feasibility of UHF-MRI for capturing individual neurobiological correlates of mental diseases. These could serve as a tool for therapy monitoring and pave the way for a truly individualized and predictive clinical approach in psychiatric care.

## Introduction

Mental disorders affect a significant proportion of the world’s population, resulting in a considerable personal, social and economic burden. Between 1990 and 2007, the rates for mental disorders increased by 31.6% and this trend continued in the period between 2007 and 2017 (percentage change in counts: 13.5%) [1]. This high level of prevalence contradicts the general upswing in neuroscience and the increasing knowledge about the neurobiological correlates of mental disorders and inevitably leads one to question the cause of this discrepancy. In this context, a view has emerged within the psychiatric community that the goal of improving care for patients with mental illness might best be achieved through a “precision medicine”, as is already the case in some somatic disciplines [2]. Consequently, there have been growing efforts to identify biomarkers suitable to base clinical decisions more specifically on the individual characteristics of each patient. In particular, great expectations have been placed on neuroimaging, whose developments over the last quarter-century have, in the opinion of many, changed the field of psychiatry considerably [3]. However, despite significant methodological advances in all areas of neuroimaging, the urgently needed clinical transfer in favor of better care for people with mental illness has, so far, failed to appear. Moreover, the American Psychiatric Association (APA) recently published a position paper stating that, up to now, neuroimaging has brought no benefit to the diagnosis and treatment of psychiatric disorders [4].

In this work we address the view that the latest developments of ultra-high field (UHF) neuroimaging technologies (defined as ≥7 T) offer further, hitherto largely unexploited opportunities for the very precise characterization of the particularities of individual patients, potentially resulting in groundbreaking progress in the development of precision psychiatry. To date, the number of UHF magnetic resonance imaging scanners (UHF-MRI) operating at 7 T, which represent the most widely used systems [5], exceeds 70 worldwide [6]. At the same time UHF-MRI at 7 T is becoming available not only for research but also for clinical use as numerous commercially available models have achieved regulatory certifications [6]. The main benefits of UHF technology include increased spatial sampling in the native image and thus a high spatial resolution, higher signal-to-noise ratio [7], higher sensitivity [8], enhanced amplitude and percent of signal change [9, 10], significantly accentuated microvasculature contributions [11], and significantly reduced nonspecific mapping signals from large vessels. These factors significantly increase the quantity of data acquired per individual scan and allow a separate consideration of single subjects with all their individual characteristics.

Here, we use the wealth of data thus obtained for a more targeted investigation of the individual organization of brain networks (NW). To date, numerous findings have indicated that a dynamic, adaptable brain NW configuration in response to one’s environment underlies healthy brain functions, and many mental disorders are now increasingly understood as network disorders. Moreover, it has been suggested that brain NWs may be potential biomarkers for research into brain-related disorders [12, 13].

One well-established method to characterize NW properties is the graph theory approach, where a NW is considered as a graph containing a set of objects, or nodes, connected by edges or links [14]. Thereby, the often-calculated graph measures include: measures of centrality (used to capture the importance of a node in a network; e.g., the node degree (D) and the betweenness centrality (BC)), measures of functional integration (e.g., global efficiency (GE) and the average path length (APL)) and measures of functional segregation (allowing the identification of subnetworks involved in specialized information processing and the assessment of their performance; e.g., clustering coefficient (CC) and local efficiency (LE)).

Although the UHF technology offers considerable advantages both in the areas of structural and functional neuroimaging [15], here we primarily focus on graph analysis of the functional NW. Considering the significantly improved signal strength, we postulate that even within a relatively short resting-state UHF-MRI acquisition, a sufficient amount of data can be collected to derive a unique connectivity pattern for the individual patient within the framework of a profound analysis. This individual connectivity profile represents a highly specific signature for each person but is also dependant on the current state of their (mental) health. Thus, it will change noticeably in a person-specific way during the course of treatment. It is anticipated that a dedicated assessment of its modifications can be brought into relation with clinical observations in order to gain valuable insights into the individual neurobiological basis of the symptoms in each patient.

In this work we show the results of such an investigation, carried out on two depressive patients in a naturalistic design. The results demonstrate the feasibility of this specific research approach in order to pave the way for a translational use of the individual connectivity patterns in everyday clinical care.

## Methods

Following the recommendations of the Declaration of Helsinki, written and informed consent was obtained from all participants. All methods were performed according to the relevant guidelines and regulations. The Ethics Committee of the Medical Faculty of the RWTH Aachen University approved the procedures of this investigation. The assessment of the patient’s global functioning was performed by the responsible clinicians using the Clinical Global Impressions scale (CGI) [16].

### Patient 1

The 54 years old male was admitted to the hospital due to depressive symptoms, mainly characterized by loss of drive and vitality, reduction of appetite, depressed mood, amnestic deficits, concentration problems and impairment in the speed of formal train of thoughts (CGI-severity score (CGI-S): 5—markedly ill).

The patient was in the fourth episode of a recurrent depressive disorder, first diagnosed seven years ago.

The first 7 T scan was conducted 13 days after hospital admission. Due to a previous successful treatment with electro-convulsion-therapy (ECT) (the last being four years ago), an ECT treatment was started again after the first 7 T scan. The patient´s daily medication included venlafaxine (225 mg), quetiapine (100 mg), quetiapine extended-release (200 mg), and lithium (30.5 mmol). The second scan was performed after 48 days. In the meantime, nine ECT treatments were performed and the lithium treatment was discontinued. At the timepoint of the second scan the patient was borderline mentally ill (CGI-S: 1; CGI-I: 1–2).

### Patient 2

The 37 years old female was admitted with the following diagnoses according to the International Statistical Classification of Diseases, 10th Revision ((ICD-10-2019): severe episode of a recurrent depressive disorder, bulimia nervosa, and emotionally unstable personality disorder, borderline typus (CGI-S: 6—Severely ill). This was the seventh psychiatric hospital admission (first: 11 years ago, last one four years ago). The medication consisted of fluoxetine (30 mg), aripiprazole (5 mg), chlorprothixen (120 mg), and pregabaline (150 mg). At the request of the patient, this medication was not changed. The treatment focus was on a psychotherapeutic intervention according to the dialectical behavioral therapy concept [17].

The first scan was performed 15 days after admission. The second scan was conducted 15 days after the first scan. At the timepoint of the second scan, the patient was mildly ill (CGI-S:3; CGI-I:2).

### MR data acquisition

The MR data were acquired using a 7 T MAGNETOM Terra scanner (Siemens Healthineers, Erlangen, Germany) equipped with a 1Tx 32Rx head coil (single channel transmit / 32-channel receive) for radiofrequency transmission and signal reception (Nova Medical, Wilmington, MA, USA).

Anatomical images were acquired with a T_1_ weighted MP-RAGE sequence within a scan time of 9 m and 15 s. Parameters were set as follows: matrix size 256 × 256 × 192 (to achieve a 0.8 mm isotropic resolution in the respective field of view (FOV)); short echo time (TE): 2.27 ms; long repetition time (TR): 4500 ms; inversion time (TI): 1000 ms; flip angle: 4°.

The resting-state fMRI data were acquired using spin-echo planar imaging (EPI) with an echo and repetition time (TE/TR) of 25 ms/2200 ms, respective. A total of 273 fMRI volumes were acquired within a 10.05 min acquisition time, with 36 slices at a slice thickness of 3.1 mm. The image matrix size was 64 × 64 and the FOV was 200 × 200 mm [2], resulting in a 3.1 mm isotropic resolution. Subjects were instructed to lie in the scanner with their eyes closed and think of nothing specific.

### Network topological changes - Non-parametric statistical approach

In order to assess subject level network topological changes, the resting-state fMRI data were first pre-processed and denoised using CONN v20.b [18], following which an ROI-ROI bivariate correlation model was applied to the data. 132 ROIs covering the whole brain were used for this purpose, which comprised cortical and subcortical parcellations from the Harvard-oxford atlas and cerebellar parcellations from the Automated Anatomical Labeling (AAL) atlas. The correlation matrices were thresholded at 0.5 (>0.5 or < -0.5) to construct the networks. The p-values of the correlations were calculated by obtaining the T-value for r(0.5) and the N(~273) of each scan. The calculated p-values were: patient 1 pre - 5.17e-17, patient 1 post – 4.33e-18, patient 2 pre – 5.64e-19, patient 2 post – 5.64e-19. The graph theory metrics of global efficiency (GE), local efficiency (LE), average path length (APL), betweenness centrality (BC), clustering coefficient (CC) and degree (D) were calculated for each ROI and imported into Matlab (Mathworks, R2020b). In order to find regions that showed a significant change post treatment, a non-parametric statistical approach similar to permutation testing was applied. First, the probability distribution of each metric in an individual’s brain network was obtained. This was done by fitting a kernel distribution to the values of a metric across ROIs in an individual’s baseline scan. The required variance and subsequently the null model were generated by drawing two samples from this distribution at random and taking their difference 100 000 times. These null models represent the probability of obtaining a difference in a certain metric at random. The p-values for each of our observed differences between the post-treatment and the baseline scans were then calculated using these null models as the fraction of tests that turned out more extreme than our observed values. Due to the conservative nature of the non-parametric approach and the fact that it corrects for a large amount of randomness, we applied a less conservative FDR correction of 0.1 to further correct for the multiple comparisons of the investigated ROIs. Supplementary Figs. S1 and S2 show the null models, the corresponding q-value (FDR corrected p-value) and p-value thresholds (in green) and the ROIs that were found to be significant as per the thresholds for patient 1 and 2, respectively.

In the second step of the analysis, an exploratory approach was used to investigate whether the significant regions showed a deviation in network properties compared to other regions in the baseline measurement; and whether these were normalized following treatment. In order to achieve this the raw values of the baseline and post-treatment scans were plotted on stem plots along with the lines representing ×1, ×2, and ×3 standard deviation, and were visually inspected.

## Results

**Fig. 1:**
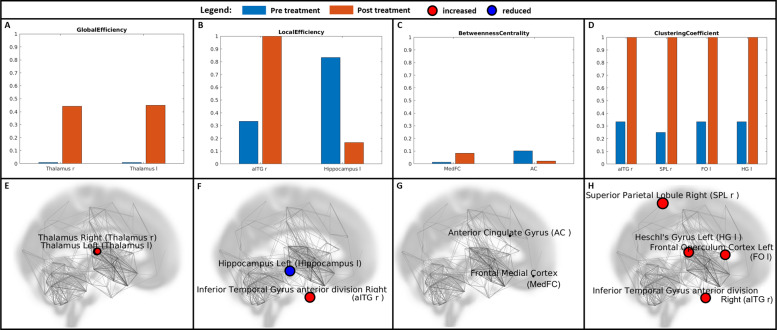
Individual changes in the network properties in patient 1. Individual changes in the network properties in patient 1 are shown as bar plots (**A**–**D**) and as network depiction (**E**–**H**) for the graph measures for which a significant change could be detected in the second measurement compared to the first one: Global Efficiency (**A**, **E**), Local Efficiency (**B**, **F**), Betweenness Centrality (**C**, **G**), Clustering Coefficient (**D**, **H**). aITGr Anterior Temporal Gyrus anterior division right, Hippocampus l Hippocampus left, MedFC Frontal Medial Cortex, AC Anterior Cingulate Gyrus, SPLr Superior Parietal Lobule right, FO l Frontal Operculum left, HG l Heschl’s Gyrus left.

**Fig. 2:**
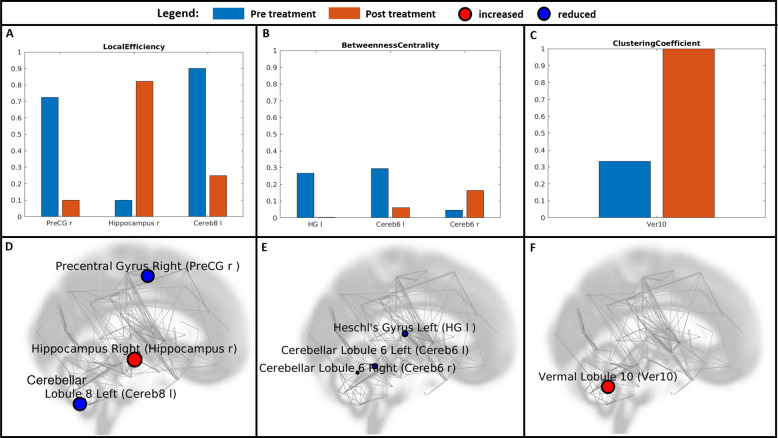
Individual changes in the network properties in patient 2. Individual changes in the network properties in patient 2 are shown as bar plots (**A**–**C**) and as a network depiction (**D**–**F**) for the graph measures for which a significant change could be detected in the second measurement compared to the first one: Local Efficiency (**A**, **D**), Betweenness Centrality (**B**, **E**), Clustering Coefficient (**C**, **F**). PreCG r Precentral Gyrus Right, Hippocampus r Hippocampus right, Cereb8 l Cerebellum 8 left, HG l Heschl’s Gyrus left, Cereb6 l Cerebellum6 left, Cereb6 r Cerebellum6 right, Ver10 Vermal Lobule 10.

The direct comparison of the different graph measures between the first and the second scan (before and after the treatment period) at an individual level revealed significant changes (q < 0.1) in several regions in both patients (Figs. 1 and 2).

For patient 1, the results of the second scan showed a significant increase in the GE of the left (q-value: 0.0707) and right (q-value: 0.0789) thalami, an increase in the LE of the right anterior inferior temporal gyrus (q-value: 0.0690) but a reduction in the LE of the left hippocampus (q-value: 0.0690). The BC of the frontal medial cortex was increased (q-value: 0.0972) and that of the anterior cingulate was found to be reduced (q-value: 0.0545) in the second scan. The CCs of four regionsn namely the right anterior inferior temporal gyrus (q-value: 0.0994), right superior parietal lobule (q-value: 0.0508), left frontal opercular cortex (0.0994) and the left Heschl’s gyrus (0.0994) increased significantly. No significant differences were observed in APL or D.

For patient 2, the results of the second scan showed a reduction in the LE of the right precentral gyrus (q-value: 0.0758) and the left cerebellar lobulus 8 (q-value: 0.0628), and an increase in the right hippocampus (q-value: 0.0330). The BC of the left Heschl’s gyrus (q-value: 0.0321) and left cerebellar lobulus 6 (q-value: 0.0585) decreased while that of the right cerebellar lobule 6 (0.0720) increased. The CC of the 10th vermal lobule also increased (0.0899). The GE, APL and D showed no significant differences.

**Fig. 3:**
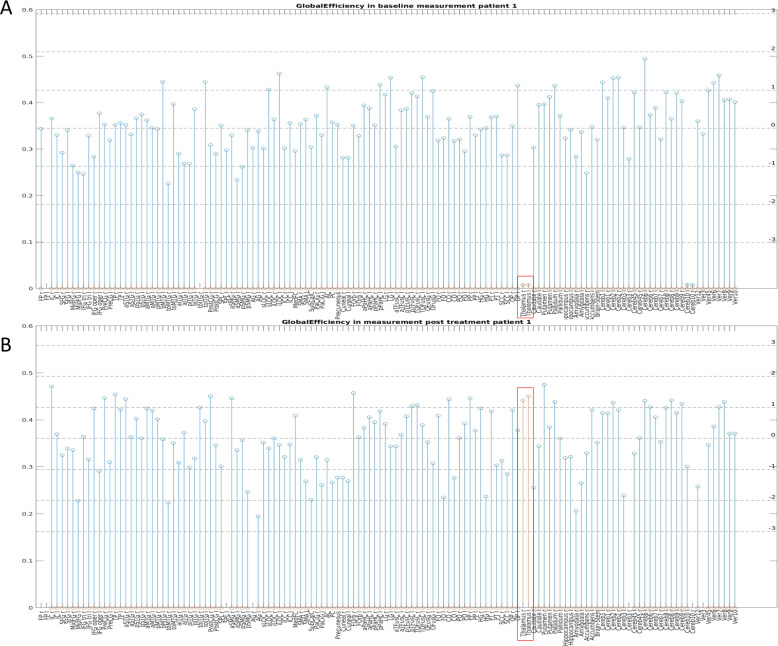
Global Efficiency (GE) across all brain regions in patient 1. **A**: GE in patient 1 during the first (pre-treatment) scan. **B**: GE in patient 1 during the second (post-treatment) scan.

**Fig. 4:**
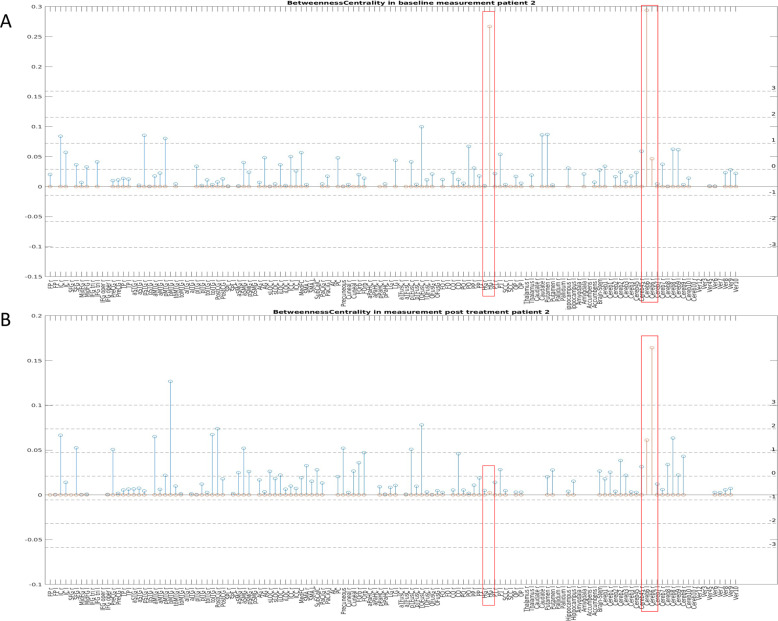
Betweenness Centrality (BC) across all brain regions in patient 2. **A:** BC in patient 2 during the first (pre-treatment) scan. **B:** BC in patient 2 during the second (post-treatment) scan.

In the second step of the analysis, we used a further exploratory approach to examine the regions showing a significant change after treatment in the first step, as explained in the methods above. Two such example stem plots are shown in Figs. 3 and 4. The observed change in the above-mentioned regions was always greater than two standard deviations. Although the values for some regions normalized after the treatment (converged to the mean value of all regions), in other regions, we observed a further movement away from the mean value, which could indicate some kind of balancing mechanism in the overall organization of the network. This can be observed from patient 2 in Fig. 4, wherein the BC of the HG and the left cerebellum 6 become normalized, but that of the right cerebellum 6 increases to more than three times the standard deviation. All values for all individual parameters measured in all regions before and after treatment are given in the supplementary table ST1.

## Discussion

Changes in functional network properties were clearly evident in the resting-state networks of both patients following a 2-week and a 6-week treatment period. In both cases, the second scan was performed at a time when the patients stated a significant improvement in their condition. Both were discharged shortly after the scan.

### Patient 1

The main finding in patient 1 was a significant increase in GE in both thalami. GE is a measure of the efficiency of the capacity for parallel information transfer, and it is typically interpreted as a measure of integrated processing [19]. An overall reduction in GE has been reported in depressive patients [20]. The human thalamus serves as a global hub, connected with the entire cortex, relaying sensory information to the cortex and mediating the transmission of cortico-cortical information [21]. Thus, the thalamus could be indicated as a critical integrative hub for functional brain networks engaged with multiple cognitive functions [22]. Different thalamic functional abnormalities have been reported in depression, with both hyperconnectivity and hypoconnectivity [23]. In our patient, an extremely reduced GE was already clearly evident in both thalami in the first (baseline) examination, indicating impaired integrative ability before the treatment. The alignment of GE in the thalami to the other regions over the course of treatment may reflect a restoration of the integrative function of the thalamus in terms of an improved ability to combine different information.

The second significant finding was a reduction of BC in the ACC. Furthermore, the BC value noticeably overreached the BC values in all other regions in the first measurement. BC quantifies the number of times that a node acts as a bridge along the shortest path between two other nodes [24]; thus, it indicates the influence of the node on the spread of information through the network [25]. The ACC is a key region of the default mode network (DMN) and an integrative hub for human socially-driven interactions, decision-making, error detection, and outcome monitoring [26]. Activity abnormalities in different parts of the ACC have been demonstrated in depression including increased functional connectivity with several brain regions [27]. Interestingly, a robust association between increased resting-state ACC activity and better treatment outcomes has been demonstrated [28]. Elevated ACC resting activity appears to foster adaptive influence on self-referential processing and an effect on the recalibration of the relationships between the DMN and a task-positive network that comprises dorsolateral prefrontal and dorsal cingulate regions implicated in cognitive control [28]. The reduction of the BC value in the course of treatment, on the other hand, could be an expression of a decrease in the influence and involvement of this region within the context of the decrease in depressive thoughts, threat monitoring and the tendency to ruminate.

In contrast to the ACC, the BC increased significantly in the frontal medial cortex (MFC), whose placement in the Conn atlas corresponds to the ventromedial prefrontal cortex (vmPFC). The vmPFC is a region involved in a variety of social, cognitive, and affective functions that are commonly disrupted in mental illness [29]. It is also known to be particularly important for value-based decision making [30] and reward [31] as well as for emotional processing [32] and regulation [33]. Consistent with our findings, a number of studies have demonstrated changes in vmPFC function as a potential marker of treatment efficacy with regard to both treatment with antidepressants [34] and psychotherapy [35, 36]. Moreover, several previous investigations have demonstrated that the efficacies of distinct treatment modalities (transcranial magnetic stimulation, electroconvulsive therapy, antidepressant medication, cognitive-behavioral therapy) can be predicted using different neuroimaging markers of vmPFC function prior to treatment initiation [29].

Furthermore, a significant increase in the CC was observed in four regions: the right inferior temporal gyrus anterior division, the right superior parietal lobule, the left frontal operculum cortex and the left Heschl’s gyrus. The inferior temporal gyrus is known for its involvement in multiple high-cognitive functions [37]. Furthermore, it exhibits extensive connections with the limbic areas [38] and thus appears to be involved in emotion regulation [39]. Similarly, the superior parietal lobule plays a pivotal role in many cognitive, perceptive, and motor-related processes [40]. As a part of attention and executive control networks, it is involved in coordinating attention under competing conditions and voluntary orienting of attention [41] positively linked to emotion regulation [42]. The frontal operculum cortex is mainly known for its function in speech production [43], but it is also involved in gustatory perception [44] and is activated during music perception [45]. Disturbed connectivity has been reported in the cingulo-opercular network in first-episode medication-naive patients with major depressive disorder [46]. Furthermore, Rolls and colleagues recently reported increased functional connectivity in the inferior frontal gyrus [47] of which the frontal operculum is a part. The authors suggested that the high functional connectivity in the right inferior frontal gyrus seen in depression could be related to a disinclination to initiate actions and may manifest as a lack of motivated action [47]. Generally, the CC reflects the tendency that neighbors of a node are also neighbors to each other [48]. Thus, the increased CC in the different regions observed here may reflect an improved segregation and optimized functioning during the course of treatment as opposed to the initially increased connectivity in depression. Indeed, lower CC has also been previously reported in depressed patients, suggesting a less specialized or segregated topological organization [49], which improved during the treatment course.

Our next finding included some divergent results regarding the LE. LE is a measure of the average efficiency of information transfer within local subgraphs or neighborhoods and is defined as the inverse of the shortest average path length of all neighbors for a given node among themselves [50]. Interestingly, previous investigations have revealed oppositely directed disturbances in the local efficiency corresponding to nodal efficiency in affective processing regions and regions involved in cognitive control in depressed patients [51]. Similarly, we observed a significant reduction in LE in the left hippocampus while seeing an increase in the right inferior temporal gyrus, a region more extensively involved in high-cognitive functions [37]. This finding corresponds with a restoration of a more favorable balance between the cognitive control and affective processing systems in parallel with the improvement of the depressive symptoms.

### Patient 2

For patient 2, the BC decreased significantly in the HG and in the left lobulus 6 of the cerebellum. The HG (Brodmans area 41/42) is a part of the temporal lobe (temporal transverse gyrus) and represents the putative anatomical correlate of the human primary auditory cortex [52]. As the temporal lobe receives some of the highest-density serotonergic innervation in the telencephalon, several investigations indicate that abnormalities in serotonergic tone in this region may be associated with depression [53]. Moreover, converging evidence suggests impaired auditory information processing in depression and reduced connectivity between the auditory cortex and the thalamus as well as the posterior cingulate [54]. The cerebellum 6 is involved in motor and in nonmotor processes (attentional/executive and default-mode) [55], including social behavior and emotional processing [56]. Furthermore, lateral parts of lobule 6 are affiliated to the salience network [57], meaning that this region may contribute to estimating the valence of salient emotional cues and in selecting appropriate behavioral responses [57]. Together, the reduced BC in the HG and cerebellum 6 following treatment indicates that they act less as bridging nodes in the post-treatment condition and have a reduced involvement in the spread of information through the network after successful treatment.

In contrast to the left region of cerebellum 6, the right region showed a significant increase in the BC value. This opposite course is difficult to explain and requires further investigations, but it may be related to the lateralization that is generally found in the cerebellum [58].

Additionally, a significant increase in the CC in the vermis 10 was observed in patient 2, as was also the case in patient 1 in several regions. The cerebellum is traditionally associated with motor control, posture coordination, and linguistic processing. However, some evidence highlights the importance of the cerebellum for higher-order functions, such as working memory, language, social and emotional task processing [55]. The Lobulus 10 (flocculonodular lobe) is a part of the oldest portion of the cerebellum (archicerebellum/vestibulocerebellum) and appears to be primarily involved in ocular motor learning [59] and in some non-motor processes [55]. In addition to vestibular functions, activations in lobule 10 have been linked to representations of dorsal attention and frontoparietal networks [60]. One other theory [61] suggests the inclusion of the lobules and vermis 9 and 10 along with the fastigial and globose nuclei into what is called the ‘limbic cerebellum’—emphasizing its role in the fight or flight response and emotion affect. Such involvement of lobule10 in emotion processing has been confirmed in a large cohort fMRI study [60]. Furthermore, an association between decreased gray matter volume in the flocculonodular lobe and depression has also been reported [62]. The increase in CC in the flocculonodular lobe indicates a higher segregation and specialization of a single region and is possibly an expression of its elevated moderation effect on emotion processing, which may contribute to a more appropriate emotion processing following treatment.

In addition to this finding, the LE decreased in one further posterior part of the cerebellum (cerebellum 8) that has generally been implicated in emotional processing [63]. It is also tightly connected to the amygdala [56] as a part of the recently described cerebello-amygdaloid network [64], with a hypothesized function for bottom-up sensory processing involving emotional evaluation, motivational appraisal, and motor preparation [64].

The LE further decreased significantly in the right precentral gyrus, that is known as the location of the primary motor cortex [65]. However, a recent investigation also revealed the involvement of this region in emotion regulation [66] and showed increased overall connectivity in patients with a major depressive disorder compared to healthy controls [67, 68]. This may reflect a compensatory process related to the psychomotor retardation observed in depressed patients [68].

In contrast to patient 1, where we found a significant decrease in LE in the left hippocampus, in patient 2, the LE increased significantly in the right hippocampus. Previous investigations have reported a distinct involvement of the left and right hippocampus in the verbal episodic and spatial memory [69] and a distinct activation depending on the aversiveness of situations and the anxiety level [70]. In addition, the hippocampal lateralization is also influenced by age [71] and gender [72]. In the context of treating patients with all the particularities of their personal characteristics, resources and difficulties, these differently directed results firmly underline the need for a close, individual examination of each patient in order to understand the exact neurobiological background of the symptomatology.

### Common remarks and outlook

In our study, two UHF-MRI measurements were performed in each of two patients, with the intention of capturing changes in the properties of the functional networks at an individual level.

Although it is not possible to state with final certainty that the changes shown here are an effect of the therapeutic intervention, coherence can be inferred based on the consistency of the results with previous findings. Moreover, the parameters in which significant changes were detected were already diverging in both patients in the initial examinations.

At this point, it should also be noted that we deliberately refrained from inter-individual comparisons and also from using control subjects in our study, as we were not interested in testing the efficacy of the therapy methods. The ultimate goal of this project was to verify whether the use of 7T-UHF-MRI in the routine clinical practice is an informative way to detect subtle changes in network properties within the context of regular treatment. The demonstrated results represent a first feasibility investigation that needs further confirmation. However, this approach hosts great potential for precision psychiatry. Until now, there have been no objective parameters for mental diseases that can be used to decide which therapy/medication is the most suitable for an individual patient. According to our approach, individual UHF-MRI scans may be a useful tool whit which to capture such parameters. For example, with a first (baseline) scan at the beginning of treatment, the deviating connectivity patterns could be recorded in an unloaded resting-state examination. A second scan could be performed after the first (test) dose of the drug or right at the beginning of other therapeutic interventions to predict the potential success of the therapy from the start. Indeed, alterations in brain connectivity have been detected using a 3 T fMRI as early as two hours after a single dose of antidepressant [73]. Therefore, an early trend towards an alignment of parameters that were noticeably deviant in the initial session could be indicative for a probable therapeutic success. On the other hand, the absence of changes in these parameters would be a warning that a different treatment pathway should be considered. The higher field strengths make it possible to study these subtle but prognosis-relevant changes at the individual level. It is anticipated that further monitoring during the course of therapy—by means of neuroimaging, but also especially in combination with detailed clinical characterization, will allow the generation of predictive algorithms that can be used to choose the most promising therapy after the initial examination. Thereby, the data shown here do not exhaust the possibilities of UHF technology by far. For example, through the inclusion of additional protocols, it is possible to capture high resolution structural data, spectroscopic data revealing concentrations of specific neurotransmitters, information about tissue energy metabolism (phosphorus ^31^P spectroscopy) as well as details relating to the cellular metabolism and tissue electrolyte homeostasis (brain sodium (^23^Na) imaging). These potentially illuminating investigations are not easily resolved at lower field strengths [74–76] but may be combined within a single 7T-UHF-MRI scan. At this point, it should also be stated that the handling and interpretation of such a huge amount of data generated in this way will be a significant challenge and one which will be best met with the help of newly emerging artificial intelligence methods.

To date, various factors have meant that the goal of identifying individual biomarkers in psychiatry has not been achievable. An individual approach using UHF-MRI could address some of those factors:

Firstly, mental diseases involve a very high variability of symptoms, and across all diagnostic categories, it is almost impossible to find two patients with exactly the same symptom profile, combined with equal mental strains on the one side and equally pronounced resources on the other side. Despite this high diversity, the knowledge about the neurobiological basis of mental illnesses is mainly based on findings that result from evaluations of averaged data from specific groups. The requirement for this strategy has arisen primarily from the low SNR of MRI systems with lower field strengths resulting in small quantities of data per individual that could only be validly analyzed at a group level [77]. Although group average designs are valuable for understanding the overall pathophysiology of specific diseases, this approach leaves a large proportion of individual information unaccounted for. Using UHF-MRI, it is now possible to detect disease-specific neurobiological alterations in a single individual. The inter-individual differences can thus be brought into relation with the symptomatology and other (medical and demographic) parameters very precisely without any further loss of information that would occur in the context of a grouping of patients.

Secondly, the eligibility requirements in most studies usually exclude relevant psychiatric comorbidities. However, clinical experience suggests high comorbidity rates in a large proportion of psychiatric patients. In a recent examination, mental comorbidities have been found in 64% of patients with mild depression and up to 78% in patients with severe depression [78]. The clinical implications of comorbidities rates range from generally greater symptom severity to difficulties in determining the adequate therapy and thereby worsened longitudinal course and poorer outcome. Also, symptoms of comorbid diseases often manifest at a subclinical level (e.g., in personality accentuation) and are not captured at all, despite being relevant to the severity of the psychic impairments and their course. Thus, the extensive characterization of patients with different mental comorbidities is an important prerequisite for understanding the special features of complex cases and the development of precisely tailored treatment strategies. Individual studies using UHF technology will allow patients to be examined in a naturalistic setting without the need to form homogeneous groups from which relevant comorbidities must be excluded.

Another point that is difficult to cover using classical group-level research is pharmacological effects, and in particular, the phenomenon of combination therapy and polypharmacy. The gold standard of clinical research is randomized clinical trials in which the effect of an investigational drug is compared with a placebo and, where relevant, the corresponding neurobiological correlates of this treatment are investigated. However, a considerable number of patients do not respond adequately to a monotherapy [79], and augmentation and/or combination strategies are commonly applied in routine clinical care to improve treatment response [80, 81]. Indeed, in a recent multicentre, multinational, cross‐sectional study, 60.64% of all participants with a major depressive disorder received augmentation and/or combination strategies with a mean number of 2.18 ± 1.22 simultaneously prescribed psychiatric drugs [82]. Against this background, precision psychiatry cannot leave such a large proportion of treatment cases unattended when evaluating the optimal individual treatment strategies. On the other hand, many studies report the neurobiological commonalities of patients with very different medication. The results are then often attributed to the shared disease pattern, assuming that the signal change related to a drug is low. Nevertheless, pharmaco-fMRI studies show consistent and reproducible effects of medication on disease relevant networks, and moreover, show that such changes may be detected even prior to clinical alterations [83]. Furthermore, in order to make a statement about the effects of a drug on the course of the disease and the associated changes at the neurobiological level, it is not sufficient to know only the mechanism of action and the dosage of the drug. Due to the genetically based individual differences in pharmacokinetics, the efficacy and side-effect profiles of individual medicines can vary considerably [84]. In the sum, the complex psychopharmacological effects on the symptomatology can only be captured using an individual approach.

Finally, patients differ considerably in terms of individual vulnerability and resources, which modulate both the severity of clinical symptoms as well as the response to treatment to a substantial extent. These parameters, in their natural complexity and interactions, can hardly be investigated in a controlled study design, and their influence can only be mapped individually.

Summing up, the central prerequisite for understanding the enormous complexity of mental illness at the neurobiological level and for a successful clinical transfer is an individualized approach. Thus, we argue that the use of UHF imaging technology, applied to study individual patients in a naturalistic treatment context, can make a valuable contribution in this regard. The new knowledge gained in this way could enable a significant extension of already existing knowledge and thus consolidate the basis for the further development of precision psychiatry.

## Supplementary information

Supplemenatry material patient 1

Supplementary material patient 2

Supplementary S1 patient 1

Supplementary S1 patient 2

## Data Availability

The code is available on request from the corresponding author for scientific use.
