# Supplementary material for: 7T ultra-high-field neuroimaging for mental health: an emerging tool for precision psychiatry?
Source: Transl Psychiatry. 2022 Jan 26;12:36. doi: 10.1038/s41398-022-01787-3 (PMC8791951; doi:10.1038/s41398-022-01787-3)
Supplement: Supplementary file 1 — Supplemenatry material patient 1 [file 41398_2022_1787_MOESM1_ESM.pdf]

Patient 1

| | Global Efficiency_pre |
|---|---|
| **'Frontal Pole Right'** | 0,344 |
| **'Frontal Pole Left'** | NaN |
| **'Insular Cortex Right'** | 0,366 |
| **'Insular Cortex Left'** | 0,330 |
| **'Superior Frontal Gyrus Right'** | 0,293 |
| **'Superior Frontal Gyrus Left'** | 0,341 |
| **'Middle Frontal Gyrus Right'** | 0,265 |
| **'Middle Frontal Gyrus Left'** | 0,250 |
| **'Inferior Frontal Gyrus pars triangularis Right'** | 0,246 |
| **'Inferior Frontal Gyrus pars triangularis Left'** | 0,328 |
| **'Inferior Frontal Gyrus pars opercularis Right'** | 0,284 |
| **'Inferior Frontal Gyrus pars opercularis Left'** | 0,377 |
| **'Precentral Gyrus Right'** | 0,353 |
| **'Precentral Gyrus Left'** | 0,319 |
| **'Temporal Pole Right'** | 0,352 |
| **'Temporal Pole Left'** | 0,356 |
| **'Superior Temporal Gyrus anterior division Right'** | 0,352 |
| **'Superior Temporal Gyrus anterior division Left'** | 0,332 |
| **'Superior Temporal Gyrus posterior division Right'** | 0,366 |
| **'Superior Temporal Gyrus posterior division Left'** | 0,375 |
| **'Middle Temporal Gyrus anterior division Right'** | 0,362 |
| **'Middle Temporal Gyrus anterior division Left'** | 0,346 |
| **'Middle Temporal Gyrus posterior division Right'** | 0,343 |
| **'Middle Temporal Gyrus posterior division Left'** | 0,445 |
| **'Middle Temporal Gyrus temporooccipital part Right'** | 0,225 |
| **'Middle Temporal Gyrus temporooccipital part Left'** | 0,397 |
| **'Inferior Temporal Gyrus anterior division Right'** | 0,290 |
| **'Inferior Temporal Gyrus anterior division Left'** | 0,269 |
| **'Inferior Temporal Gyrus posterior division Right'** | 0,268 |
| **'Inferior Temporal Gyrus posterior division Left'** | 0,386 |
| **'Inferior Temporal Gyrus temporooccipital part Right'** | NaN |
| **'Inferior Temporal Gyrus temporooccipital part Left'** | 0,445 |
| **'Postcentral Gyrus Right'** | 0,309 |
| **'Postcentral Gyrus Left'** | 0,290 |
| **'Superior Parietal Lobule Right'** | 0,351 |
| **'Superior Parietal Lobule Left'** | 0,298 |
| **'Supramarginal Gyrus anterior division Right'** | 0,330 |
| **'Supramarginal Gyrus anterior division Left'** | 0,234 |
| **'Supramarginal Gyrus posterior division Right'** | 0,262 |
| **'Supramarginal Gyrus posterior division Left'** | 0,340 |
| **'Angular Gyrus Right'** | 0,303 |
| **'Angular Gyrus Left'** | 0,339 |
| **'Lateral Occipital Cortex superior division Right'** | 0,302 |
| **'Lateral Occipital Cortex superior division Left'** | 0,429 |
| **'Lateral Occipital Cortex inferior division Right'** | 0,364 |
| **'Lateral Occipital Cortex inferior division Left'** | 0,463 |
| **'Intracalcarine Cortex Right'** | 0,302 |
| **'Intracalcarine Cortex Left'** | 0,356 |
| **'Frontal Medial Cortex'** | 0,296 |
| **'Supplementary Motor Cortex- Right'** | 0,354 |
| **'Supplementary Motor Cortex- Left'** | 0,364 |
| **'Subcallosal Cortex'** | 0,304 |

| | |
|---|---|
| **'Paracingulate Gyrus Right'** | 0,372 |
| **'Paracingulate Gyrus Left'** | 0,330 |
| **'Anterior Cingulate Gyrus'** | 0,433 |
| **'Posterior Cingulate Gyrus'** | 0,358 |
| **'Precuneus Cortex'** | 0,353 |
| **'Cuneal Cortex Right'** | 0,281 |
| **'Cuneal Cortex Left'** | 0,281 |
| **'Frontal Orbital Cortex Right'** | 0,351 |
| **'Frontal Orbital Cortex Left'** | 0,328 |
| **'Parahippocampal Gyrus anterior division Right'** | 0,394 |
| **'Parahippocampal Gyrus anterior division Left'** | 0,388 |
| **'Parahippocampal Gyrus posterior division Right'** | 0,351 |
| **'Parahippocampal Gyrus posterior division Left'** | 0,439 |
| **'Lingual Gyrus Right'** | 0,418 |
| **'Lingual Gyrus Left'** | 0,454 |
| **'Temporal Fusiform Cortex anterior division Right'** | 0,306 |
| **'Temporal Fusiform Cortex anterior division Left'** | 0,384 |
| **'Temporal Fusiform Cortex posterior division Right'** | 0,387 |
| **'Temporal Fusiform Cortex posterior division Left'** | 0,421 |
| **'Temporal Occipital Fusiform Cortex Right'** | 0,414 |
| **'Temporal Occipital Fusiform Cortex Left'** | 0,455 |
| **'Occipital Fusiform Gyrus Right'** | 0,370 |
| **'Occipital Fusiform Gyrus Left'** | 0,425 |
| **'Frontal Operculum Cortex Right'** | 0,319 |
| **'Frontal Operculum Cortex Left'** | 0,324 |
| **'Central Opercular Cortex Right'** | 0,365 |
| **'Central Opercular Cortex Left'** | 0,318 |
| **'Parietal Operculum Cortex Right'** | 0,321 |
| **'Parietal Operculum Cortex Left'** | 0,295 |
| **'Planum Polare Right'** | 0,370 |
| **'Planum Polare Left'** | 0,330 |
| **'Heschl''s Gyrus Right'** | 0,342 |
| **'Heschl''s Gyrus Left'** | 0,346 |
| **'Planum Temporale Right'** | 0,369 |
| **'Planum Temporale Left'** | 0,370 |
| **'Supracalcarine Cortex Right'** | 0,287 |
| **'Supracalcarine Cortex Left'** | 0,287 |
| **'Occipital Pole Right'** | 0,350 |
| **'Occipital Pole Left'** | 0,437 |
| **'Thalamus Right'** | 0,008 |
| **'Thalamus Left'** | 0,008 |
| **'Caudate Right'** | 0,303 |
| **'Caudate Left'** | 0,395 |
| **'Putamen Right'** | 0,397 |
| **'Putamen Left'** | 0,412 |
| **'Pallidum Right'** | 0,436 |
| **'Pallidum Left'** | 0,372 |
| **'Hippocampus Right'** | 0,324 |
| **'Hippocampus Left'** | 0,342 |
| **'Amygdala Right'** | 0,284 |
| **'Amygdala Left'** | 0,337 |
| **'Accumbens Right'** | 0,248 |
| **'Accumbens Left'** | 0,348 |

Patient 1

| | |
|---|---|
| **'Brain-Stem'** | 0,320 |
| **'Cerebellar Crus1 Left'** | 0,444 |
| **'Cerebellar Crus1 Right'** | 0,410 |
| **'Cerebellar Crus2 Left'** | 0,453 |
| **'Cerebellar Crus2 Right'** | 0,454 |
| **'Cerebellar Lobule 3 Left'** | 0,346 |
| **'Cerebellar Lobule 3 Right'** | 0,279 |
| **'Cerebellar Lobule 4 and 5 Left'** | 0,422 |
| **'Cerebellar Lobule 4 and 5 Right'** | 0,347 |
| **'Cerebellar Lobule 6 Left'** | 0,494 |
| **'Cerebellar Lobule 6 Right'** | 0,373 |
| **'Cerebellar Lobule 7b Left'** | 0,389 |
| **'Cerebellar Lobule 7b Right'** | 0,322 |
| **'Cerebellar Lobule 8 Left'** | 0,422 |
| **'Cerebellar Lobule 8 Right'** | 0,365 |
| **'Cerebellar Lobule 9 Left'** | 0,421 |
| **'Cerebellar Lobule 9 Right'** | 0,403 |
| **'Cerebellar Lobule 10 Left'** | 0,008 |
| **'Cerebellar Lobule 10 Right'** | 0,008 |
| **'Vermal Lobule 1 and 2'** | 0,360 |
| **'Vermal Lobule 3'** | 0,332 |
| **'Vermal Lobule 4 and 5'** | 0,426 |
| **'Vermal Lobule 6'** | 0,443 |
| **'Vermal Lobule 7'** | 0,459 |
| **'Vermal Lobule 8'** | 0,406 |
| **'Vermal Lobule 9'** | 0,408 |
| **'Vermal Lobule 10'** | 0,401 |

| Local Efficiency_pre | Betweenness Centrality_pre | Average Path Length_pre | Clustering coefficient_pre |
|---|---|---|---|
| 0,167 | 0,023 | 3,064 | 0,167 |
| NaN | NaN | NaN | NaN |
| 0,897 | 0,020 | 3,160 | 0,795 |
| 0,536 | 0,016 | 3,384 | 0,357 |
| 0,600 | 0,003 | 3,776 | 0,600 |
| 0,633 | 0,015 | 3,208 | 0,600 |
| 0,833 | 0,000 | 4,144 | 0,667 |
| 1,000 | NaN | 4,224 | 1,000 |
| NaN | 0,000 | 4,472 | NaN |
| 1,000 | NaN | 3,216 | 1,000 |
| 0,500 | 0,005 | 3,984 | 0,500 |
| 0,718 | 0,015 | 2,912 | 0,472 |
| 0,537 | 0,051 | 3,312 | 0,364 |
| 0,267 | 0,030 | 3,448 | 0,267 |
| 0,841 | 0,008 | 3,480 | 0,686 |
| 0,602 | 0,018 | 3,064 | 0,357 |
| 0,865 | 0,006 | 3,480 | 0,733 |
| 0,800 | 0,005 | 3,208 | 0,600 |
| 0,897 | 0,020 | 3,160 | 0,795 |
| 0,651 | 0,026 | 3,000 | 0,429 |
| 0,738 | 0,028 | 3,392 | 0,610 |
| 0,478 | 0,021 | 3,216 | 0,333 |
| 0,897 | 0,003 | 3,504 | 0,795 |
| 0,713 | 0,036 | 2,624 | 0,456 |
| 1,000 | NaN | 4,632 | 1,000 |
| 0,785 | 0,007 | 2,848 | 0,578 |
| 0,333 | 0,002 | 3,664 | 0,333 |
| NaN | NaN | 3,864 | NaN |
| 0,833 | 0,003 | 3,992 | 0,667 |
| 0,536 | 0,023 | 2,872 | 0,357 |
| NaN | NaN | NaN | NaN |
| 0,752 | 0,040 | 2,600 | 0,522 |
| 0,869 | 0,004 | 3,776 | 0,750 |
| 0,333 | 0,007 | 3,752 | 0,333 |
| 0,452 | 0,044 | 3,144 | 0,250 |
| NaN | 0,008 | 3,496 | NaN |
| 0,922 | 0,002 | 3,544 | 0,844 |
| NaN | NaN | 4,440 | NaN |
| 1,000 | NaN | 3,992 | 1,000 |
| 0,350 | 0,006 | 3,184 | 0,300 |
| 0,733 | 0,005 | 3,608 | 0,500 |
| 0,750 | 0,003 | 3,192 | 0,500 |
| 0,717 | 0,008 | 3,672 | 0,500 |
| 0,697 | 0,035 | 2,608 | 0,436 |
| 0,843 | 0,001 | 3,176 | 0,694 |
| 0,701 | 0,066 | 2,472 | 0,433 |
| 0,500 | 0,003 | 3,752 | 0,500 |
| 0,403 | 0,032 | 3,320 | 0,361 |
| 0,667 | 0,013 | 3,648 | 0,400 |
| 0,522 | 0,027 | 3,088 | 0,400 |
| 0,800 | 0,005 | 2,912 | 0,600 |
| 1,000 | NaN | 3,448 | 1,000 |

| | | | |
|---|---|---|---|
| 0,588 | 0,041 | 3,024 | 0,309 |
| 0,833 | 0,006 | 3,320 | 0,667 |
| 0,500 | 0,101 | 2,584 | 0,238 |
| 0,565 | 0,022 | 3,120 | 0,389 |
| 0,470 | 0,040 | 3,128 | 0,286 |
| 0,500 | 0,017 | 3,840 | 0,500 |
| 0,917 | 0,002 | 3,888 | 0,833 |
| 0,797 | 0,017 | 3,464 | 0,736 |
| 0,650 | 0,012 | 3,320 | 0,400 |
| 0,352 | 0,040 | 2,784 | 0,194 |
| 0,641 | 0,052 | 2,856 | 0,378 |
| 0,533 | 0,014 | 3,176 | 0,400 |
| 0,716 | 0,025 | 2,808 | 0,471 |
| 0,804 | 0,006 | 2,944 | 0,608 |
| 0,754 | 0,017 | 2,736 | 0,510 |
| 0,333 | 0,002 | 3,480 | 0,333 |
| 0,584 | 0,039 | 2,872 | 0,311 |
| 0,411 | 0,030 | 2,872 | 0,357 |
| 0,781 | 0,013 | 2,776 | 0,571 |
| 0,748 | 0,010 | 2,984 | 0,503 |
| 0,744 | 0,024 | 2,728 | 0,493 |
| 0,882 | 0,000 | 3,176 | 0,764 |
| 0,794 | 0,007 | 2,792 | 0,588 |
| 0,800 | 0,010 | 3,744 | 0,782 |
| 0,478 | 0,023 | 3,512 | 0,333 |
| 0,852 | 0,013 | 3,256 | 0,703 |
| 0,800 | 0,003 | 3,400 | 0,600 |
| 0,917 | 0,001 | 3,624 | 0,833 |
| 0,333 | 0,003 | 3,648 | 0,333 |
| 0,879 | 0,023 | 3,152 | 0,758 |
| 0,762 | 0,004 | 3,320 | 0,524 |
| 0,897 | 0,004 | 3,528 | 0,795 |
| 0,400 | 0,013 | 3,152 | 0,333 |
| 0,844 | 0,018 | 3,248 | 0,695 |
| 0,379 | 0,040 | 3,064 | 0,222 |
| 0,850 | 0,003 | 3,864 | 0,700 |
| 0,850 | 0,003 | 3,864 | 0,700 |
| 0,783 | 0,001 | 3,152 | 0,600 |
| 0,730 | 0,037 | 2,664 | 0,478 |
| NaN | NaN | 1,000 | NaN |
| NaN | NaN | 1,000 | NaN |
| NaN | 0,001 | 3,408 | NaN |
| 0,125 | 0,077 | 2,712 | 0,107 |
| 0,446 | 0,085 | 2,744 | 0,429 |
| 0,620 | 0,059 | 2,640 | 0,345 |
| 0,481 | 0,059 | 2,544 | 0,264 |
| 0,786 | 0,011 | 2,864 | 0,571 |
| 1,000 | NaN | 3,464 | 1,000 |
| 0,833 | 0,004 | 3,416 | 0,667 |
| 1,000 | NaN | 3,656 | 1,000 |
| 0,778 | 0,005 | 3,192 | 0,600 |
| NaN | NaN | 4,192 | NaN |
| 0,518 | 0,027 | 3,200 | 0,357 |

| | | | |
|---|---|---|---|
| 0,722 | 0,001 | 3,464 | 0,500 |
| 0,779 | 0,009 | 2,736 | 0,558 |
| 0,648 | 0,050 | 2,832 | 0,560 |
| 0,774 | 0,011 | 2,712 | 0,547 |
| 0,720 | 0,021 | 2,696 | 0,460 |
| 0,476 | 0,005 | 3,368 | 0,429 |
| 0,833 | 0,000 | 4,064 | 0,667 |
| 0,645 | 0,024 | 2,920 | 0,404 |
| 0,900 | 0,001 | 3,240 | 0,800 |
| 0,694 | 0,066 | 2,528 | 0,419 |
| 0,871 | 0,000 | 3,168 | 0,742 |
| 0,827 | 0,004 | 3,072 | 0,654 |
| 0,333 | 0,008 | 3,288 | 0,333 |
| 0,795 | 0,010 | 2,872 | 0,595 |
| 0,722 | 0,011 | 3,136 | 0,667 |
| 0,829 | 0,003 | 2,960 | 0,658 |
| 0,816 | 0,004 | 3,064 | 0,632 |
| NaN | NaN | 1,000 | NaN |
| NaN | NaN | 1,000 | NaN |
| 0,756 | 0,006 | 3,336 | 0,561 |
| 0,802 | 0,002 | 3,488 | 0,619 |
| 0,732 | 0,046 | 2,768 | 0,581 |
| 0,776 | 0,016 | 2,736 | 0,562 |
| 0,774 | 0,013 | 2,720 | 0,551 |
| 0,859 | 0,002 | 3,064 | 0,719 |
| 0,817 | 0,006 | 2,912 | 0,633 |
| 0,786 | 0,010 | 2,992 | 0,583 |

| Degree_pre | obal Efficiency_pc | Local Efficiency_post | etweenness Centrality_po |
|---|---|---|---|
| 4,000 | NaN | NaN | NaN |
| NaN | NaN | NaN | NaN |
| 13,000 | 0,471 | 0,787 | 0,021 |
| 8,000 | 0,370 | 0,233 | 0,028 |
| 5,000 | 0,325 | 0,800 | 0,002 |
| 6,000 | 0,339 | 0,658 | 0,019 |
| 3,000 | 0,336 | 1,000 | NaN |
| 2,000 | 0,228 | NaN | NaN |
| 2,000 | 0,364 | 0,786 | 0,004 |
| 4,000 | 0,316 | 0,540 | 0,024 |
| 4,000 | 0,425 | 0,757 | 0,014 |
| 9,000 | 0,291 | 0,850 | 0,005 |
| 11,000 | 0,447 | 0,712 | 0,034 |
| 6,000 | 0,310 | 0,400 | 0,020 |
| 15,000 | 0,454 | 0,753 | 0,018 |
| 8,000 | 0,422 | 0,570 | 0,040 |
| 15,000 | 0,444 | 0,845 | 0,005 |
| 5,000 | 0,363 | 0,750 | 0,002 |
| 13,000 | 0,402 | 0,686 | 0,020 |
| 7,000 | 0,361 | 0,661 | 0,011 |
| 15,000 | 0,425 | 0,864 | 0,004 |
| 6,000 | 0,420 | 0,730 | 0,021 |
| 13,000 | 0,402 | 0,871 | 0,009 |
| 17,000 | 0,359 | 0,389 | 0,014 |
| 2,000 | 0,224 | NaN | NaN |
| 10,000 | 0,350 | 0,433 | 0,017 |
| 3,000 | 0,309 | 1,000 | NaN |
| 1,000 | 0,373 | 0,286 | 0,018 |
| 3,000 | 0,299 | 0,333 | 0,014 |
| 8,000 | 0,318 | 0,417 | 0,005 |
| NaN | 0,426 | 0,676 | 0,039 |
| 17,000 | 0,397 | 0,527 | 0,033 |
| 8,000 | 0,450 | 0,652 | 0,055 |
| 4,000 | 0,346 | 0,400 | 0,041 |
| 8,000 | 0,301 | 1,000 | NaN |
| 2,000 | NaN | NaN | NaN |
| 10,000 | 0,446 | 0,748 | 0,020 |
| 1,000 | 0,335 | 0,405 | 0,048 |
| 2,000 | 0,357 | 0,821 | 0,002 |
| 5,000 | 0,247 | NaN | 0,014 |
| 5,000 | NaN | NaN | NaN |
| 5,000 | 0,195 | NaN | NaN |
| 5,000 | 0,352 | 0,133 | 0,034 |
| 13,000 | 0,339 | 0,167 | 0,007 |
| 9,000 | 0,360 | 0,850 | 0,005 |
| 19,000 | 0,346 | 0,400 | 0,008 |
| 4,000 | 0,321 | 0,900 | 0,002 |
| 9,000 | 0,348 | 0,601 | 0,024 |
| 5,000 | 0,409 | 0,470 | 0,083 |
| 6,000 | 0,315 | 0,333 | 0,010 |
| 5,000 | 0,269 | 0,833 | 0,000 |
| 2,000 | 0,230 | NaN | NaN |

| | | | |
|---|---|---|---|
| 11,000 | 0,321 | 0,756 | 0,009 |
| 6,000 | 0,261 | 1,000 | NaN |
| 15,000 | 0,315 | 0,500 | 0,021 |
| 9,000 | 0,267 | 0,333 | 0,006 |
| 8,000 | 0,277 | 0,750 | 0,005 |
| 4,000 | 0,277 | 0,750 | 0,005 |
| 4,000 | 0,270 | 0,333 | 0,007 |
| 14,000 | 0,458 | 0,818 | 0,011 |
| 6,000 | 0,363 | 0,642 | 0,008 |
| 9,000 | 0,383 | 0,658 | 0,013 |
| 10,000 | 0,406 | 0,641 | 0,023 |
| 6,000 | 0,395 | 0,735 | 0,009 |
| 21,000 | 0,418 | 0,813 | 0,009 |
| 18,000 | 0,392 | 0,646 | 0,024 |
| 25,000 | 0,343 | 0,833 | 0,004 |
| 3,000 | 0,344 | 0,833 | 0,001 |
| 10,000 | 0,368 | 0,300 | 0,035 |
| 8,000 | 0,408 | 0,567 | 0,040 |
| 15,000 | 0,430 | 0,742 | 0,030 |
| 18,000 | 0,432 | 0,770 | 0,017 |
| 25,000 | 0,389 | 0,773 | 0,020 |
| 11,000 | 0,353 | 0,583 | 0,004 |
| 18,000 | 0,308 | NaN | 0,000 |
| 11,000 | 0,409 | 0,850 | 0,007 |
| 6,000 | 0,235 | 1,000 | NaN |
| 14,000 | 0,443 | 0,773 | 0,034 |
| 5,000 | 0,277 | 0,333 | 0,006 |
| 9,000 | 0,362 | 0,333 | 0,022 |
| 3,000 | 0,392 | 0,286 | 0,044 |
| 14,000 | 0,447 | 0,797 | 0,012 |
| 7,000 | 0,377 | 0,374 | 0,021 |
| 13,000 | 0,424 | 0,893 | 0,003 |
| 6,000 | 0,237 | 1,000 | NaN |
| 15,000 | 0,418 | 0,843 | 0,013 |
| 10,000 | 0,303 | 0,400 | 0,013 |
| 5,000 | 0,313 | 0,867 | 0,004 |
| 5,000 | 0,285 | 0,333 | 0,010 |
| 5,000 | 0,421 | 0,495 | 0,051 |
| 17,000 | 0,378 | 0,722 | 0,007 |
| 1,000 | 0,442 | 0,789 | 0,022 |
| 1,000 | 0,450 | 0,682 | 0,048 |
| 2,000 | 0,255 | NaN | NaN |
| 8,000 | 0,344 | 0,600 | 0,016 |
| 8,000 | 0,475 | 0,767 | 0,036 |
| 11,000 | 0,384 | 0,518 | 0,014 |
| 14,000 | 0,438 | 0,757 | 0,019 |
| 7,000 | 0,360 | 0,476 | 0,029 |
| 5,000 | 0,319 | NaN | 0,005 |
| 9,000 | 0,322 | 0,167 | 0,016 |
| 2,000 | 0,206 | NaN | NaN |
| 6,000 | 0,265 | NaN | 0,014 |
| 1,000 | 0,329 | 0,917 | 0,000 |
| 8,000 | 0,421 | 0,756 | 0,016 |

| | | | |
|---|---|---|---|
| 4,000 | 0,352 | 0,804 | 0,002 |
| 22,000 | 0,415 | 0,798 | 0,007 |
| 14,000 | 0,414 | 0,658 | 0,030 |
| 24,000 | 0,437 | 0,771 | 0,015 |
| 24,000 | 0,421 | 0,722 | 0,016 |
| 7,000 | 0,239 | NaN | NaN |
| 4,000 | NaN | NaN | NaN |
| 19,000 | 0,328 | 0,300 | 0,018 |
| 6,000 | 0,362 | 0,643 | 0,008 |
| 31,000 | 0,440 | 0,731 | 0,037 |
| 12,000 | 0,427 | 0,706 | 0,027 |
| 13,000 | 0,406 | 0,792 | 0,006 |
| 3,000 | 0,353 | 0,600 | 0,002 |
| 18,000 | 0,425 | 0,790 | 0,015 |
| 9,000 | 0,442 | 0,514 | 0,070 |
| 20,000 | 0,415 | 0,763 | 0,013 |
| 17,000 | 0,434 | 0,772 | 0,016 |
| 1,000 | 0,301 | 1,000 | NaN |
| 1,000 | NaN | NaN | NaN |
| 12,000 | 0,258 | NaN | NaN |
| 7,000 | NaN | NaN | NaN |
| 17,000 | 0,347 | 0,267 | 0,031 |
| 21,000 | 0,386 | 0,624 | 0,022 |
| 26,000 | 0,427 | 0,819 | 0,012 |
| 18,000 | 0,438 | 0,753 | 0,026 |
| 16,000 | 0,371 | 0,694 | 0,015 |
| 16,000 | 0,371 | 0,642 | 0,019 |

| verage Path Length_po | lustering coefficient_po | Degree_post |
|---|---|---|
| NaN | NaN | NaN |
| NaN | NaN | NaN |
| 2,492 | 0,576 | 24,000 |
| 2,863 | 0,200 | 6,000 |
| 3,290 | 0,600 | 5,000 |
| 3,250 | 0,393 | 8,000 |
| 3,226 | 1,000 | 4,000 |
| 4,524 | NaN | 1,000 |
| 3,000 | 0,571 | 7,000 |
| 3,484 | 0,381 | 7,000 |
| 2,685 | 0,525 | 16,000 |
| 3,726 | 0,700 | 5,000 |
| 2,629 | 0,529 | 21,000 |
| 3,532 | 0,400 | 6,000 |
| 2,613 | 0,549 | 23,000 |
| 2,685 | 0,429 | 15,000 |
| 2,645 | 0,690 | 21,000 |
| 3,065 | 0,536 | 8,000 |
| 2,774 | 0,455 | 12,000 |
| 3,073 | 0,429 | 8,000 |
| 2,726 | 0,728 | 17,000 |
| 2,718 | 0,514 | 15,000 |
| 2,903 | 0,743 | 15,000 |
| 3,056 | 0,286 | 7,000 |
| 4,605 | NaN | 1,000 |
| 3,048 | 0,300 | 5,000 |
| 3,427 | 1,000 | 2,000 |
| 2,887 | 0,238 | 7,000 |
| 3,613 | 0,333 | 3,000 |
| 3,331 | 0,333 | 4,000 |
| 2,613 | 0,615 | 14,000 |
| 2,774 | 0,527 | 11,000 |
| 2,637 | 0,485 | 22,000 |
| 3,185 | 0,400 | 6,000 |
| 3,484 | 1,000 | 2,000 |
| NaN | NaN | NaN |
| 2,669 | 0,519 | 22,000 |
| 3,250 | 0,333 | 7,000 |
| 3,153 | 0,643 | 8,000 |
| 4,226 | NaN | 2,000 |
| NaN | NaN | NaN |
| 5,218 | NaN | 1,000 |
| 3,081 | 0,133 | 6,000 |
| 3,121 | 0,167 | 4,000 |
| 2,992 | 0,700 | 5,000 |
| 3,153 | 0,400 | 6,000 |
| 3,435 | 0,800 | 6,000 |
| 3,250 | 0,464 | 8,000 |
| 2,718 | 0,273 | 11,000 |
| 3,427 | 0,333 | 3,000 |
| 3,984 | 0,667 | 3,000 |
| 4,508 | NaN | 1,000 |

| | | |
|---|---|---|
| 3,411 | 0,533 | 6,000 |
| 4,081 | 1,000 | 3,000 |
| 3,516 | 0,333 | 7,000 |
| 3,976 | 0,333 | 3,000 |
| 3,871 | 0,500 | 4,000 |
| 3,871 | 0,500 | 4,000 |
| 4,000 | 0,333 | 3,000 |
| 2,565 | 0,636 | 22,000 |
| 2,935 | 0,400 | 5,000 |
| 2,879 | 0,393 | 8,000 |
| 2,742 | 0,364 | 11,000 |
| 2,871 | 0,500 | 12,000 |
| 2,750 | 0,625 | 16,000 |
| 2,903 | 0,379 | 12,000 |
| 3,258 | 0,667 | 7,000 |
| 3,113 | 0,667 | 4,000 |
| 2,952 | 0,200 | 6,000 |
| 2,758 | 0,318 | 12,000 |
| 2,645 | 0,533 | 16,000 |
| 2,685 | 0,549 | 18,000 |
| 2,919 | 0,712 | 12,000 |
| 3,218 | 0,429 | 8,000 |
| 3,508 | NaN | 3,000 |
| 2,790 | 0,714 | 14,000 |
| 4,468 | 1,000 | 2,000 |
| 2,645 | 0,568 | 20,000 |
| 3,863 | 0,333 | 3,000 |
| 3,048 | 0,333 | 7,000 |
| 2,742 | 0,238 | 7,000 |
| 2,653 | 0,602 | 22,000 |
| 3,016 | 0,244 | 10,000 |
| 2,710 | 0,787 | 17,000 |
| 4,444 | 1,000 | 2,000 |
| 2,702 | 0,705 | 15,000 |
| 3,605 | 0,400 | 5,000 |
| 3,548 | 0,733 | 6,000 |
| 3,758 | 0,333 | 3,000 |
| 2,669 | 0,308 | 13,000 |
| 2,992 | 0,467 | 10,000 |
| 2,661 | 0,579 | 20,000 |
| 2,516 | 0,484 | 18,000 |
| 4,016 | NaN | 1,000 |
| 3,145 | 0,600 | 5,000 |
| 2,476 | 0,547 | 25,000 |
| 2,839 | 0,500 | 8,000 |
| 2,694 | 0,537 | 20,000 |
| 3,024 | 0,476 | 7,000 |
| 3,242 | NaN | 2,000 |
| 3,315 | 0,167 | 4,000 |
| 4,919 | NaN | 1,000 |
| 3,927 | NaN | 2,000 |
| 3,266 | 0,833 | 4,000 |
| 2,710 | 0,533 | 15,000 |

| | | |
|---|---|---|
| 3,161 | 0,643 | 8,000 |
| 2,887 | 0,596 | 19,000 |
| 2,815 | 0,450 | 16,000 |
| 2,726 | 0,548 | 21,000 |
| 2,831 | 0,480 | 19,000 |
| 4,339 | NaN | 1,000 |
| NaN | NaN | NaN |
| 3,347 | 0,300 | 5,000 |
| 3,097 | 0,536 | 8,000 |
| 2,702 | 0,486 | 21,000 |
| 2,831 | 0,443 | 21,000 |
| 2,879 | 0,592 | 16,000 |
| 3,065 | 0,600 | 5,000 |
| 2,726 | 0,588 | 17,000 |
| 2,524 | 0,314 | 15,000 |
| 2,742 | 0,533 | 15,000 |
| 2,694 | 0,550 | 19,000 |
| 3,573 | 1,000 | 2,000 |
| NaN | NaN | NaN |
| 4,048 | NaN | 1,000 |
| NaN | NaN | NaN |
| 3,097 | 0,267 | 6,000 |
| 3,065 | 0,429 | 15,000 |
| 2,702 | 0,640 | 17,000 |
| 2,629 | 0,516 | 18,000 |
| 2,935 | 0,476 | 7,000 |
| 3,056 | 0,473 | 11,000 |
