# Supplementary material for: 7T ultra-high-field neuroimaging for mental health: an emerging tool for precision psychiatry?
Source: Transl Psychiatry. 2022 Jan 26;12:36. doi: 10.1038/s41398-022-01787-3 (PMC8791951; doi:10.1038/s41398-022-01787-3)
Supplement: Supplementary file 2 — Supplementary material patient 2 [file 41398_2022_1787_MOESM2_ESM.pdf]

Patient 2

	Global Efficiency_pre	ocal Efficiency_pr
'Frontal Pole Right'	0,208	0,333
'Frontal Pole Left'	0,285	1,000
'Insular Cortex Right'	0,433	0,684
'Insular Cortex Left'	0,422	0,741
'Superior Frontal Gyrus Right'	0,151	1,000
'Superior Frontal Gyrus Left'	0,357	0,746
'Middle Frontal Gyrus Right'	0,177	0,333
'Middle Frontal Gyrus Left'	0,344	0,439
'Inferior Frontal Gyrus pars triangularis Right'	NaN	NaN
'Inferior Frontal Gyrus pars triangularis Left'	0,220	NaN
'Inferior Frontal Gyrus pars opercularis Right'	0,173	1,000
'Inferior Frontal Gyrus pars opercularis Left'	0,173	NaN
'Precentral Gyrus Right'	0,358	0,725
'Precentral Gyrus Left'	0,326	0,653
'Temporal Pole Right'	0,375	0,791
'Temporal Pole Left'	0,373	0,786
'Superior Temporal Gyrus anterior division Right'	NaN	NaN
'Superior Temporal Gyrus anterior division Left'	0,332	0,857
'Superior Temporal Gyrus posterior division Right'	0,306	0,400
'Superior Temporal Gyrus posterior division Left'	0,304	0,850
'Middle Temporal Gyrus anterior division Right'	0,265	0,333
'Middle Temporal Gyrus anterior division Left'	0,298	0,601
'Middle Temporal Gyrus posterior division Right'	0,254	0,333
'Middle Temporal Gyrus posterior division Left'	0,251	1,000
'Middle Temporal Gyrus temporooccipital part Right'	0,303	0,800
'Middle Temporal Gyrus temporooccipital part Left'	NaN	NaN
'Inferior Temporal Gyrus anterior division Right'	NaN	NaN
'Inferior Temporal Gyrus anterior division Left'	0,011	NaN
'Inferior Temporal Gyrus posterior division Right'	0,212	0,333
'Inferior Temporal Gyrus posterior division Left'	0,275	0,833
'Inferior Temporal Gyrus temporooccipital part Right'	0,264	0,722
'Inferior Temporal Gyrus temporooccipital part Left'	0,249	0,722
'Postcentral Gyrus Right'	0,329	0,730
'Postcentral Gyrus Left'	0,380	0,780
'Superior Parietal Lobule Right'	0,281	0,642
'Superior Parietal Lobule Left'	0,203	1,000
'Supramarginal Gyrus anterior division Right'	0,309	0,730
'Supramarginal Gyrus anterior division Left'	0,343	0,622
'Supramarginal Gyrus posterior division Right'	0,371	0,740
'Supramarginal Gyrus posterior division Left'	0,241	1,000
'Angular Gyrus Right'	0,181	0,333
'Angular Gyrus Left'	0,353	0,686
'Lateral Occipital Cortex superior division Right'	0,261	0,833
'Lateral Occipital Cortex superior division Left'	0,266	0,167
'Lateral Occipital Cortex inferior division Right'	0,241	NaN
'Lateral Occipital Cortex inferior division Left'	0,198	NaN
'Intracalcarine Cortex Right'	0,294	0,533
'Intracalcarine Cortex Left'	0,264	0,683
'Frontal Medial Cortex'	0,286	0,429
'Supplementary Motor Cortex- Right'	0,307	0,587
'Supplementary Motor Cortex- Left'	0,247	1,000
'Subcallosal Cortex'	0,261	1,000

'Paracingulate Gyrus Right'	0,292	0,750
'Paracingulate Gyrus Left'	0,330	0,690
'Anterior Cingulate Gyrus'	0,241	1,000
'Posterior Cingulate Gyrus'	0,369	0,544
'Precuneus Cortex'	0,261	0,833
'Cuneal Cortex Right'	0,230	0,833
'Cuneal Cortex Left'	0,205	0,833
'Frontal Orbital Cortex Right'	0,322	0,732
'Frontal Orbital Cortex Left'	0,178	NaN
'Parahippocampal Gyrus anterior division Right'	NaN	NaN
'Parahippocampal Gyrus anterior division Left'	0,136	NaN
'Parahippocampal Gyrus posterior division Right'	0,207	0,833
'Parahippocampal Gyrus posterior division Left'	0,242	0,417
'Lingual Gyrus Right'	0,249	1,000
'Lingual Gyrus Left'	0,307	0,367
'Temporal Fusiform Cortex anterior division Right'	0,011	NaN
'Temporal Fusiform Cortex anterior division Left'	0,015	NaN
'Temporal Fusiform Cortex posterior division Right'	0,232	NaN
'Temporal Fusiform Cortex posterior division Left'	0,244	NaN
'Temporal Occipital Fusiform Cortex Right'	0,310	0,381
'Temporal Occipital Fusiform Cortex Left'	0,284	0,333
'Occipital Fusiform Gyrus Right'	0,306	0,500
'Occipital Fusiform Gyrus Left'	0,185	NaN
'Frontal Operculum Cortex Right'	0,248	NaN
'Frontal Operculum Cortex Left'	0,147	NaN
'Central Opercular Cortex Right'	0,393	0,732
'Central Opercular Cortex Left'	0,358	0,788
'Parietal Operculum Cortex Right'	0,338	0,778
'Parietal Operculum Cortex Left'	0,406	0,742
'Planum Polare Right'	0,401	0,762
'Planum Polare Left'	0,382	0,787
'Heschl''s Gyrus Right'	0,344	0,889
'Heschl''s Gyrus Left'	0,411	0,647
'Planum Temporale Right'	0,363	0,793
'Planum Temporale Left'	0,336	0,405
'Supracalcarine Cortex Right'	0,230	0,833
'Supracalcarine Cortex Left'	0,205	0,833
'Occipital Pole Right'	0,197	NaN
'Occipital Pole Left'	0,173	NaN
'Thalamus Right'	0,190	NaN
'Thalamus Left'	0,244	NaN
'Caudate Right'	0,237	1,000
'Caudate Left'	0,320	0,375
'Putamen Right'	0,348	0,600
'Putamen Left'	0,348	0,865
'Pallidum Right'	NaN	NaN
'Pallidum Left'	NaN	NaN
'Hippocampus Right'	0,262	0,100
'Hippocampus Left'	0,197	NaN
'Amygdala Right'	0,197	NaN
'Amygdala Left'	0,315	0,500
'Accumbens Right'	0,237	1,000
'Accumbens Left'	0,276	0,833

**'Brain-Stem'**	0,190	NaN
**'Cerebellar Crus1 Left'**	0,239	NaN
**'Cerebellar Crus1 Right'**	0,185	NaN
**'Cerebellar Crus2 Left'**	0,173	NaN
**'Cerebellar Crus2 Right'**	0,191	NaN
**'Cerebellar Lobule 3 Left'**	0,258	0,750
**'Cerebellar Lobule 3 Right'**	0,248	0,417
**'Cerebellar Lobule 4 and 5 Left'**	0,299	0,650
**'Cerebellar Lobule 4 and 5 Right'**	0,329	0,599
**'Cerebellar Lobule 6 Left'**	0,394	0,501
**'Cerebellar Lobule 6 Right'**	0,303	0,533
**'Cerebellar Lobule 7b Left'**	0,288	0,800
**'Cerebellar Lobule 7b Right'**	0,229	NaN
**'Cerebellar Lobule 8 Left'**	0,267	0,900
**'Cerebellar Lobule 8 Right'**	0,305	0,606
**'Cerebellar Lobule 9 Left'**	0,336	0,676
**'Cerebellar Lobule 9 Right'**	0,274	0,800
**'Cerebellar Lobule 10 Left'**	0,161	NaN
**'Cerebellar Lobule 10 Right'**	0,239	1,000
**'Vermal Lobule 1 and 2'**	0,190	NaN
**'Vermal Lobule 3'**	NaN	NaN
**'Vermal Lobule 4 and 5'**	0,303	0,867
**'Vermal Lobule 6'**	0,281	0,833
**'Vermal Lobule 7'**	0,142	NaN
**'Vermal Lobule 8'**	0,319	0,694
**'Vermal Lobule 9'**	0,335	0,730
**'Vermal Lobule 10'**	0,293	0,417

›etweenness Centrality_pr‹verage Path Length_pr‹lustering coefficient_p			Degree_pre
0,020	5,208	0,333	3,000
NaN	3,900	1,000	2,000
0,084	2,958	0,397	26,000
0,057	2,983	0,482	23,000
NaN	7,125	1,000	2,000
0,036	3,475	0,500	13,000
0,007	6,158	0,333	3,000
0,033	3,542	0,273	11,000
NaN	NaN	NaN	NaN
0,041	5,158	NaN	3,000
NaN	6,167	1,000	2,000
NaN	6,150	NaN	1,000
0,010	3,558	0,486	15,000
0,011	3,675	0,528	9,000
0,014	3,158	0,582	14,000
0,013	3,258	0,575	16,000
NaN	NaN	NaN	NaN
0,002	3,408	0,714	8,000
0,085	3,542	0,400	5,000
0,000	3,600	0,700	5,000
0,018	4,100	0,333	3,000
0,022	4,117	0,464	8,000
0,080	4,275	0,333	4,000
NaN	4,383	1,000	2,000
0,005	3,583	0,600	5,000
NaN	NaN	NaN	NaN
NaN	NaN	NaN	NaN
NaN	1,500	NaN	1,000
0,034	5,200	0,333	4,000
0,002	4,175	0,667	4,000
0,011	4,442	0,500	4,000
0,003	4,592	0,500	4,000
0,008	3,717	0,491	11,000
0,013	3,250	0,562	18,000
0,001	4,292	0,400	5,000
NaN	5,417	1,000	2,000
0,001	3,892	0,476	7,000
0,040	3,633	0,379	12,000
0,024	3,508	0,507	17,000
NaN	4,608	1,000	2,000
0,007	6,150	0,333	4,000
0,048	3,483	0,424	12,000
0,000	4,375	0,667	3,000
0,005	4,300	0,167	4,000
0,037	4,475	NaN	3,000
0,002	5,400	NaN	2,000
0,050	3,733	0,400	6,000
0,026	4,242	0,429	7,000
0,057	4,217	0,381	7,000
0,003	3,967	0,476	7,000
NaN	4,550	1,000	3,000
NaN	4,375	1,000	3,000

0,004	4,192	0,536	8,000
0,017	3,625	0,417	9,000
NaN	4,550	1,000	2,000
0,048	3,458	0,358	16,000
0,000	4,375	0,667	3,000
0,004	4,683	0,667	4,000
0,000	5,217	0,667	3,000
0,020	3,650	0,714	8,000
0,014	6,133	NaN	2,000
NaN	NaN	NaN	NaN
NaN	7,475	NaN	1,000
0,000	5,300	0,667	3,000
0,005	4,558	0,333	4,000
NaN	4,275	1,000	3,000
0,044	3,642	0,333	6,000
NaN	1,500	NaN	1,000
0,000	1,000	NaN	2,000
0,041	4,550	NaN	2,000
0,004	4,458	NaN	3,000
0,100	3,608	0,286	7,000
0,011	3,742	0,333	3,000
0,021	3,433	0,500	4,000
NaN	5,558	NaN	1,000
0,012	4,300	NaN	2,000
NaN	7,125	NaN	1,000
0,024	3,308	0,476	21,000
0,012	3,258	0,582	11,000
0,005	3,625	0,591	12,000
0,067	3,058	0,505	20,000
0,031	3,092	0,526	20,000
0,018	3,192	0,574	17,000
0,002	3,358	0,778	10,000
0,267	2,808	0,426	17,000
0,022	3,250	0,603	13,000
0,054	3,350	0,333	7,000
0,004	4,683	0,667	4,000
0,000	5,217	0,667	3,000
0,017	5,383	NaN	2,000
0,006	6,208	NaN	2,000
NaN	5,300	NaN	1,000
0,019	4,308	NaN	3,000
NaN	4,517	1,000	3,000
0,086	3,542	0,321	8,000
0,087	3,408	0,556	10,000
0,003	3,525	0,731	13,000
NaN	NaN	NaN	NaN
NaN	NaN	NaN	NaN
0,031	4,333	0,100	5,000
NaN	5,325	NaN	1,000
NaN	5,325	NaN	1,000
0,021	3,433	0,500	4,000
NaN	4,517	1,000	3,000
0,007	3,975	0,667	4,000

0,028	5,508	NaN	2,000
0,034	4,567	NaN	4,000
NaN	5,558	NaN	1,000
0,016	6,358	NaN	3,000
0,024	5,617	NaN	2,000
0,008	4,375	0,500	5,000
0,018	4,458	0,333	4,000
0,024	3,725	0,400	6,000
0,059	3,525	0,333	10,000
0,294	2,900	0,242	14,000
0,046	3,667	0,400	6,000
0,004	3,892	0,600	6,000
0,037	4,725	NaN	2,000
0,000	4,275	0,800	6,000
0,062	3,817	0,444	9,000
0,061	3,483	0,400	11,000
0,003	4,200	0,600	6,000
0,014	6,483	NaN	2,000
NaN	4,467	1,000	2,000
NaN	5,450	NaN	1,000
NaN	NaN	NaN	NaN
0,001	3,667	0,733	6,000
0,001	3,783	0,667	3,000
NaN	7,350	NaN	1,000
0,023	3,592	0,444	9,000
0,028	3,483	0,473	11,000
0,022	3,708	0,333	4,000

lobal Efficiency_po	ocal Efficiency_pc	tweenness Centrality_p	verage Path Length_po
0,024	NaN	0,000	2,200
0,025	NaN	0,000	1,800
0,392	0,634	0,067	3,017
0,309	0,661	0,014	3,574
0,281	0,900	0,000	3,687
0,365	0,546	0,053	2,974
0,025	NaN	0,000	1,800
0,031	NaN	0,001	1,400
NaN	NaN	NaN	NaN
0,018	NaN	NaN	2,600
NaN	NaN	NaN	NaN
0,025	NaN	0,000	1,800
0,303	0,100	0,051	3,461
0,282	0,333	0,002	3,643
0,302	0,750	0,005	3,626
0,302	0,750	0,006	3,652
0,301	0,417	0,007	3,435
0,355	0,808	0,007	3,270
0,290	0,738	0,004	3,783
0,255	1,000	NaN	4,157
0,404	0,621	0,065	2,687
0,349	0,818	0,006	3,078
0,366	0,679	0,022	2,922
0,365	0,667	0,127	2,783
0,265	0,600	0,010	4,035
0,319	0,893	0,001	3,243
0,241	1,000	NaN	4,383
0,266	0,950	0,001	3,948
0,297	0,881	0,000	3,565
0,362	0,780	0,012	2,965
0,271	0,833	0,003	3,739
0,321	0,200	0,067	3,139
0,397	0,661	0,074	2,817
0,362	0,715	0,018	3,261
0,266	1,000	NaN	3,878
0,280	0,833	0,002	3,652
0,373	0,699	0,025	2,983
0,322	0,571	0,052	3,200
0,368	0,720	0,026	3,000
0,188	NaN	NaN	5,191
0,245	NaN	0,017	4,200
0,334	0,836	0,003	3,183
0,376	0,725	0,026	2,930
0,328	0,683	0,018	3,078
0,309	0,357	0,022	3,365
0,231	0,500	0,006	4,583
0,315	0,852	0,010	3,461
0,303	0,917	0,007	3,574
0,366	0,666	0,019	3,104
0,352	0,673	0,033	3,096
0,281	NaN	0,015	3,452
0,344	0,663	0,028	3,165

0,272	0,333	0,013	3,739
0,248	NaN	NaN	3,965
0,204	NaN	NaN	4,730
0,347	0,730	0,021	3,174
0,325	0,522	0,052	3,165
0,290	0,889	0,003	3,800
0,294	0,436	0,026	3,861
0,323	0,539	0,036	3,452
0,328	0,609	0,047	3,226
0,192	NaN	NaN	5,278
0,263	1,000	NaN	3,930
0,292	0,793	0,009	4,000
0,273	0,911	0,001	4,061
0,307	0,863	0,008	3,565
0,309	0,811	0,011	3,557
NaN	NaN	NaN	NaN
0,292	0,500	0,001	3,574
0,330	0,705	0,051	3,417
0,298	0,822	0,010	3,809
0,346	0,650	0,078	3,209
0,292	0,819	0,003	3,835
0,285	0,900	0,000	3,583
0,233	NaN	0,005	4,200
0,319	0,767	0,003	3,357
0,220	NaN	NaN	4,565
0,340	0,689	0,005	3,339
0,381	0,713	0,046	2,913
0,340	0,822	0,005	3,200
0,304	0,933	0,002	3,461
0,323	0,571	0,011	3,409
0,337	0,684	0,019	3,530
0,322	0,810	0,005	3,496
0,335	0,845	0,003	3,391
0,338	0,711	0,014	3,496
0,342	0,726	0,028	3,304
0,301	0,844	0,005	3,652
NaN	NaN	NaN	NaN
0,249	0,850	0,003	4,252
0,247	0,833	0,003	4,278
0,008	NaN	NaN	1,000
0,008	NaN	NaN	1,000
0,008	NaN	NaN	1,000
0,008	NaN	NaN	1,000
0,255	0,300	0,020	4,261
0,277	0,500	0,028	3,896
0,221	1,000	NaN	4,774
0,221	1,000	NaN	4,774
0,291	0,822	0,004	3,904
0,287	0,405	0,015	3,739
0,008	NaN	NaN	1,000
0,008	NaN	NaN	1,000
0,258	1,000	NaN	3,974
0,191	NaN	NaN	5,252

0,261	0,476	0,027	4,287
0,261	NaN	0,018	3,957
0,319	0,508	0,025	3,191
0,263	0,833	0,004	4,148
0,289	0,643	0,038	3,678
0,292	0,667	0,022	4,000
0,275	0,811	0,003	4,235
0,277	0,833	0,003	4,096
0,294	0,476	0,031	3,670
0,338	0,367	0,061	3,226
0,369	0,449	0,164	2,887
0,273	0,730	0,012	3,974
0,265	0,833	0,006	4,035
0,275	0,250	0,034	3,722
0,329	0,167	0,063	3,009
0,296	0,524	0,022	3,530
0,308	0,524	0,043	3,339
0,192	NaN	NaN	5,278
0,243	1,000	NaN	4,157
0,215	1,000	NaN	4,965
0,206	NaN	NaN	4,991
0,213	NaN	NaN	4,661
0,304	0,856	0,003	3,539
0,286	0,889	0,003	3,835
0,244	0,333	0,006	4,183
0,249	0,500	0,007	4,087
0,236	1,000	NaN	4,226

lustering coefficient_po	Degree_post
NaN	2,000
NaN	2,000
0,415	19,000
0,571	8,000
0,800	5,000
0,333	13,000
NaN	2,000
NaN	3,000
NaN	NaN
NaN	1,000
NaN	NaN
NaN	2,000
0,100	5,000
0,333	3,000
0,536	8,000
0,536	8,000
0,333	4,000
0,615	14,000
0,524	7,000
1,000	4,000
0,382	17,000
0,636	12,000
0,513	13,000
0,556	9,000
0,600	5,000
0,786	8,000
1,000	4,000
0,900	5,000
0,762	7,000
0,564	13,000
0,667	3,000
0,200	5,000
0,390	17,000
0,467	16,000
1,000	3,000
0,667	4,000
0,448	15,000
0,429	7,000
0,462	14,000
NaN	1,000
NaN	3,000
0,673	11,000
0,467	15,000
0,467	6,000
0,286	7,000
0,500	4,000
0,711	10,000
0,833	9,000
0,390	15,000
0,527	11,000
NaN	2,000
0,422	10,000

0,333	3,000
NaN	1,000
NaN	1,000
0,491	11,000
0,400	6,000
0,778	9,000
0,418	11,000
0,356	10,000
0,361	9,000
NaN	1,000
1,000	3,000
0,591	12,000
0,821	8,000
0,733	10,000
0,644	10,000
NaN	NaN
0,500	4,000
0,439	12,000
0,644	10,000
0,372	13,000
0,639	9,000
0,800	5,000
NaN	2,000
0,533	6,000
NaN	1,000
0,545	12,000
0,457	15,000
0,644	10,000
0,867	6,000
0,393	8,000
0,436	13,000
0,639	9,000
0,691	11,000
0,440	14,000
0,474	13,000
0,689	10,000
NaN	NaN
0,700	5,000
0,667	6,000
NaN	1,000
NaN	1,000
NaN	1,000
NaN	1,000
0,300	5,000
0,500	4,000
1,000	3,000
1,000	3,000
0,644	10,000
0,381	7,000
NaN	1,000
NaN	1,000
1,000	2,000
NaN	1,000

0,476	7,000
NaN	3,000
0,333	7,000
0,667	7,000
0,571	7,000
0,500	12,000
0,622	10,000
0,667	9,000
0,476	7,000
0,267	10,000
0,288	12,000
0,524	7,000
0,667	6,000
0,200	5,000
0,167	4,000
0,333	7,000
0,333	7,000
NaN	1,000
1,000	3,000
1,000	3,000
NaN	1,000
NaN	1,000
0,711	10,000
0,778	9,000
0,333	3,000
0,500	4,000
1,000	3,000
