# Supplementary figures and images for: 7T ultra-high-field neuroimaging for mental health: an emerging tool for precision psychiatry?
Source: Transl Psychiatry. 2022 Jan 26;12:36. doi: 10.1038/s41398-022-01787-3 (PMC8791951; doi:10.1038/s41398-022-01787-3)

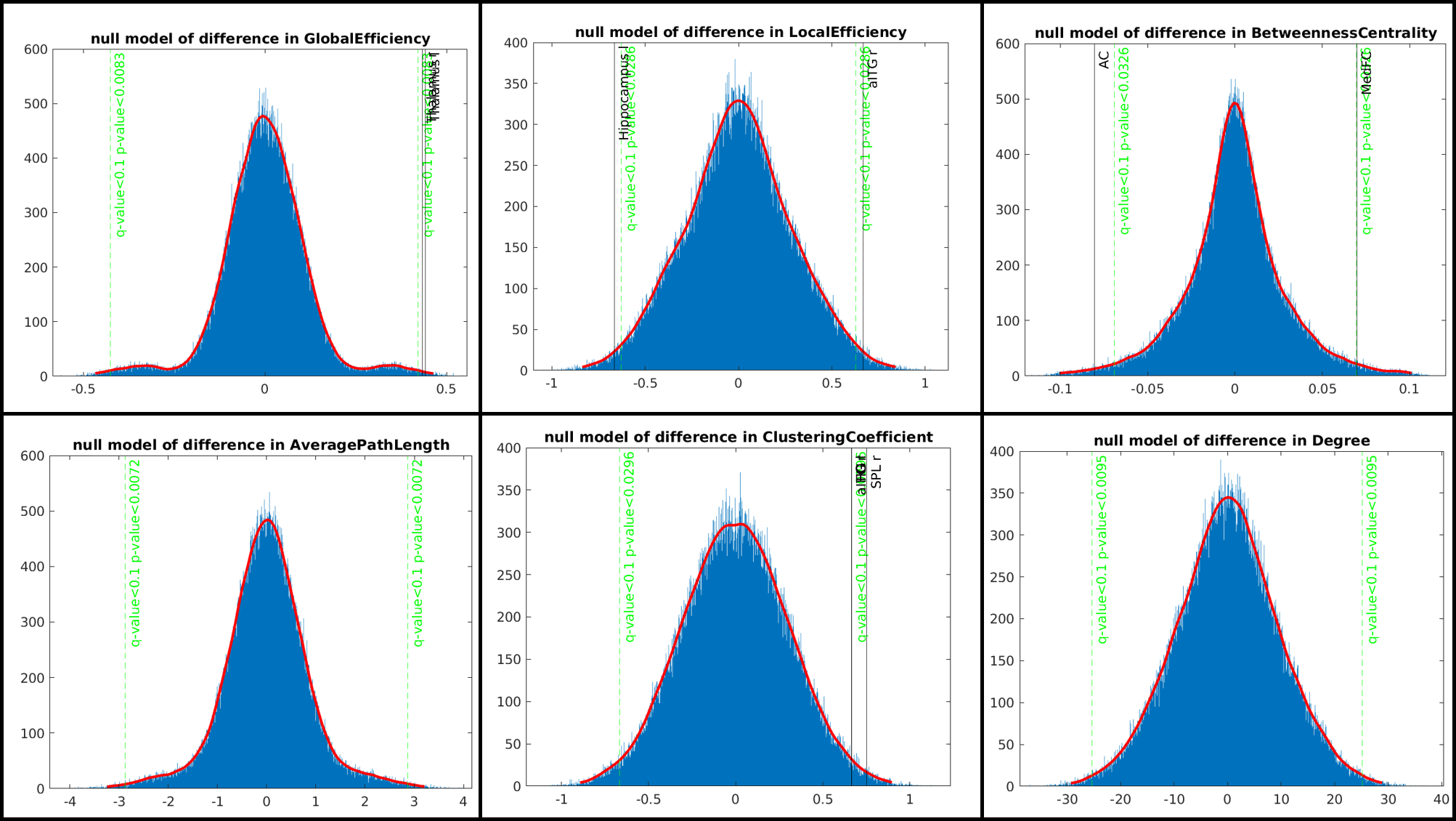

Supplement: Supplementary file 3 — Supplementary S1 patient 1 [file 41398_2022_1787_MOESM3_ESM.png]

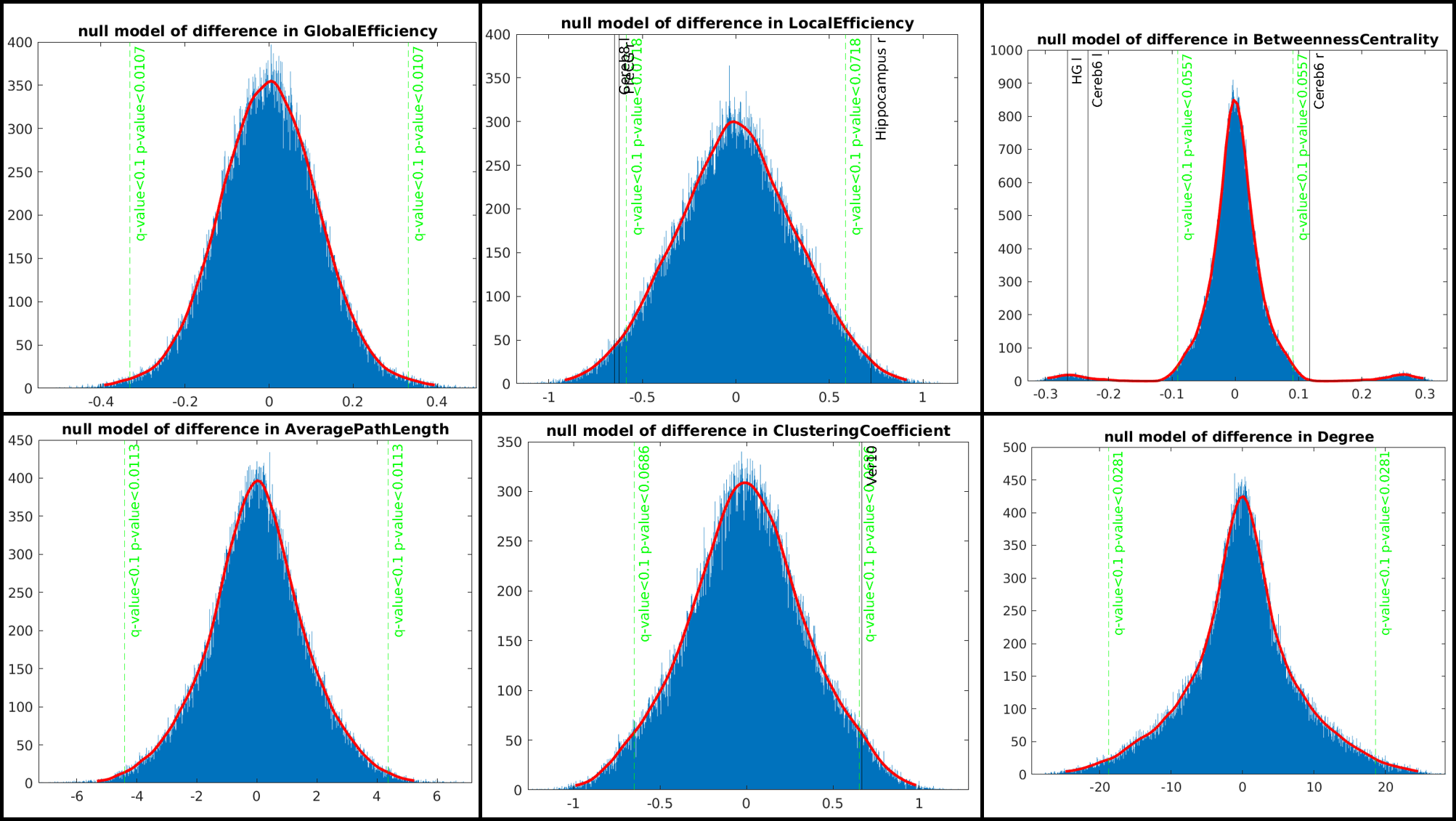

Supplement: Supplementary file 4 — Supplementary S1 patient 2 [file 41398_2022_1787_MOESM4_ESM.png]
